# Old Age-Related Stereotypes of Preschool Children

**DOI:** 10.3389/fpsyg.2020.00807

**Published:** 2020-04-28

**Authors:** Allison Flamion, Pierre Missotten, Lucie Jennotte, Noémie Hody, Stéphane Adam

**Affiliations:** Psychology of Aging Unit, Department of Psychology and Clinics of Human Systems, University of Liège, Liège, Belgium

**Keywords:** ageism, grandparents, older people, social learning, stereotype content model

## Abstract

Ageist attitudes have been discovered in children as early as 3 years. However, so far very few studies, especially during the last decade, have examined age-related stereotypes in preschool children. Available questionnaires adapted to this population are scarce. Our study was designed to probe old age-related views in 3- to 6-year-old children (*n* = 126) using both an open-ended Image-of-Aging question and a new pilot tool, called Young Children’s Views of Older People (YCVOP), based on a visual analog scale illustrated by cartoons. Parental views of older people were also collected. The YCVOP was easy to use and internally consistent. Both that scale and the Image-of-Aging question showed globally favorable views of older people in preschool children, especially regarding warmth and smartness traits. However, assessment of physical capacity and independence tended to be negative. The overall results were in line with the low-competence, high-warmth stereotype of older people that is common in young adults and school-age children and was found in parents in the current study (Stereotype Content Model). Strikingly, children’s views did not correlate with those of their parents’: The children’s responses appeared more personal and emotional, while the parents tended to adopt global stereotypes. The preschoolers’ views of older people were much more positive in those who spontaneously evoked their grandparents when asked to think of an old person. In conclusion, this study, introducing a new visual tool to assess age-related stereotypes, suggests ambivalent views of older adults start in preschool children and are influenced by grandparents relationships.

## Introduction

Ageism is defined as negative stereotypes against older people leading to discrimination, segregation, and disregard (Butler, 1969). Ageism is less known and more tolerated in our societies than other forms of discrimination like racism or sexism but it is highly prevalent (European Commission, 2015) and its consequences can be severe. For instance, older people holding and internalizing negative age stereotypes and attitudes have decreased cognitive and physical performance (Levy, 2009; Armstrong et al., 2017). There is general agreement, in our rapidly aging societies, that ageism should be combatted to foster healthier aging and better integration of older people in social activities (Officer and de la Fuente-Núñez, 2018).

Negative views and attitudes toward older adults can start early in life. When school-age children are asked what they think of the old, they often reveal negative views and ageist stereotypes. In several studies, children regarded older people as ugly and sick (Weinberger, 1979), and associated them with a decline in physical and psychological capacities such as deterioration of skin, bones, posture, hearing, and thinking power, as well as bad temper, impatience, and inability to cope with stress (Goldman and Goldman, 1981). Children also described older people as less active, weak, and slow (Lynott and Merola, 2007). All age-related stereotypes, however, were not unequivocally negative: On the contrary, they seemed in some cases similar to those expressed by young adults, who perceive lower competence but higher warmth in older vs. younger adults (Fiske et al., 2002). Warmth and competence are cardinal dimensions along which social perceptions of groups of individuals align (Fiske et al., 1999) and stereotypes content can be described [cf. the Stereotype Content Model (Fiske et al., 2002)]. Warmth can be assessed using adjectives such as kind, friendly, trustworthy, while competence is described by skillful, assertive, intelligent, independent (Cuddy et al., 2008). However, studies specifically designed to test for warmth and competence within children’s views of older people are rare. In this respect, Vauclair et al. (2018) found that 6- to 10-year-old children rated older people higher on a warmth scale than on a competence scale, whereas Balázs (2013) observed that fourth grade children gave low levels of both warmth and competence to old vs. young individuals.

School-age children can also be tested for their potential attitudes toward, and tendency to discriminate against, the older age group. For example, 6- to 7- and 9- to 11-year-olds were less likely to choose the older individual as someone they would want to spend time with (Davidson et al., 2008), and 6- to 11-year-olds had a bias toward recalling negative information about older vs. younger adults (Davidson et al., 1995). Likewise, 8- to 11-year-old children showed a strong preference for young over old persons in an implicit association test (Babcock et al., 2016).

A question that follows up naturally is: Do preschool children think and behave in the same way as school-age children? The answer to that question is important in order to understand how age-related views and attitudes develop during the early life stages, if they are balanced or plainly stereotypical, and if the low-competence, high-warmth stereotype of old adults is the rule. Such data could help build up improved interventional programs in preschool children, since the published outcomes of these programs implemented in preschoolers have not been uniformly successful as of yet (Seefeldt, 1987; Middlecamp and Gross, 2002; Bertram et al., 2018). Similarly, a systematic review showed that programs based on actual contact, or on media and instructions, and designed to reduce ethnic prejudice and discrimination in children 8 years and under gave mixed results with positive (40%), non-significant (50%), and negative (10%) effects (Aboud et al., 2012).

Studies on preschoolers’ views of older people are limited. These views have mostly been collected through the semantic differential scales of the Children’s Attitudes Toward the Elderly (CATE) questionnaire (Jantz et al., 1976), scales adapted from it, or open-ended individual interviews. Results showed that preschool children as young as 3 years consider older people as often sick, unattractive, involved in passive activities such as sitting and talking, and needing help (Seefeldt et al., 1977; Seefeldt, 1987), but also kind and friendly (Dellmann-Jenkins et al., 1986; Middlecamp and Gross, 2002; Holmes, 2009). A more recent study using classroom discussions also revealed dual views of older people, described as suffering from health challenges but being good playmates (Bertram et al., 2018). Other studies using photographs or drawings of persons of different ages observed that preschoolers systematically picked up the younger versus the older faces in terms of attractiveness and liking (Kogan et al., 1961; Weinberger, 1979), or as the more trustworthy person in case their safety and health were at stake (Miller et al., 1984). Kwong See and her group showed that 3- and 5-year-olds acted differently according to the age of the person they were dealing with: They presumed an older interlocutor was less able than a younger one to teach them new words (Kwong See and Nicoladis, 2010), and displayed age stereotypes when they interpreted the motivation of an old versus a young interlocutor asking them the same question twice (Kwong See et al., 2012).

Overall, the age-related stereotypes have not been sufficiently approached in preschool children in recent years. This paucity may be explained in large part by the lack of available questionnaires adapted to a very young population with limited linguistic abilities. One way to explore age-related stereotypes in very young children would be to use questionnaires based on pictures and administered on an individual basis. This method has provided reliable evidence in the evaluation of ethnic (Branch and Newcombe, 1986) and gender (Reis and Wright, 1982) stereotypes from 3 to 4 years on. Yet, it has seldom been used in the exploration of age-related stereotypes in preschool children. The CATE questionnaire contains hand drawn faces of different ages (including older faces) from which children have to pick up one face in closed-ended questions (e.g., “Who is the nicest?”); drawings are also used to ensure children identify old vs. young people. A small study has explored the use of seven-card picture series illustrating a bipolar adjective scale (Caspi, 1984). Overall, however, there is thus a need to assess age-related stereotypes in a larger sample of preschool children using an age-appropriate tool based on pictures or cartoons adapted to the child’s cognitive development. Developing and using such a tool will be the first goal of our study.

Another aspect that has received insufficient attention in preschool children is the analysis of factors favoring the development of negative stereotypes toward older people. In our increasingly digital era, media take a large and growing place in young children’s lives. The media representation of older people (especially older women) is often stereotypical and negative; that can influence children’s perceptions and lead to ageist stereotypes (Bergman, 2017). A second main factor in this younger age group is parental influence. According to Gilbert and Ricketts (2008), parents inspire the construction of ageist stereotypes and prejudice in early childhood, especially during the first socialization experience and learning process. Parental influence on racial and gender stereotypes has been shown in children as young as 4–5 years (Meyer, 1980; Branch and Newcombe, 1986). Degner and Dalege (2013), in their large meta-analysis of intergroup attitudes, concluded that child–parent attitudes are related throughout childhood and adolescence, becoming stronger with age. However, when the question of parental influence on children’s age-related stereotypes was examined in a recent study, the stereotypes of 6- to 7-year-olds did not correlate at all with their parents’, whereas those of 9- to 10- and 13- to 14-year-olds did (Lineweaver et al., 2017). This may set the age-related stereotypes apart from other forms of prejudice. Furthermore, to our knowledge, the correlation between child and parent age-related stereotypes has not been investigated in children younger than 6 years. This will be the second goal of our study.

Based on previous research described above, the current study aimed at creating and using an improved measure of age-related views in preschool children. Our goal was also to relate the children’s views to their parent’s beliefs. We hypothesized that preschoolers’ views would follow a stereotypical pattern evoking the doddering but dear perception (low competence, high warmth) described in young adults and in school-age children and adolescents. A second hypothesis was that there is a low or absent correlation between the preschoolers’ and their parents’ views of older people.

## Materials and Methods

### Sample

We recruited 126 children aged 3 to 6 in pre-elementary school or kindergarten from the French-speaking part of Belgium. For each child, we also invited one of her/his parents to participate in the study. There was no systematic selection of either father or mother. The selected parent filled an informed consent form for both her/his participation and that of her/his child.

### Data Collection

The children were interviewed individually at home or at school for about 30 min and their responses were noted. Their selected parent completed a two-measure questionnaire in writing at home. The entire study was conducted in French.

### Measures

#### Socio-Demographic Data

Personal data collected from the parents included their age, gender, marital status, highest level of education reached, and their child’s age.

#### Children’s Views of Older People

These views were assessed using, in sequence, an open-ended Image-of-Aging question (Flamion et al., 2019) and a novel controlled scale which we call Young Children’s Views of Older People (YCVOP).

The Image-of-Aging measure requiring short answers was: *“Give the five words that first come to your mind when you think of an old person.”* “*Old person*” was not defined in order to avoid cues that may orient the children’s responses. We asked external evaluators (*n* = 128; age range, 18–68 years) to assign a score on a Likert-type scale ranging from −5 (extremely negative) to +5 (extremely positive) to each word given in reference to an old person. Evaluators were all of Caucasian origin and French-speaking. The majority were female (∼85%), had at least undergraduate level of education (∼70%), and were current university students (∼75%). Previous studies on adults (Levy and Langer, 1994), adolescents, and children (Flamion et al., 2019) have shown this method of evaluation to be effective and reliable. In the current study, inter-rater reliability was assessed by computing intraclass correlations: the result was 0.98 (two-way random, average measures, consistency). The words with a mean score between −5 and −1 were judged as *negative*; those scored from <−0.99 to +0.99, as *neutral*; and those scored from +1 to +5, as *positive*. Using these scores, a global Image-of-Aging score was then allocated to each child: this was the weighted mean of all his word scores (some children gave only 1, 2, 3, or 4 words). The first word cited (i.e., the most spontaneous one) was given a weight of 5, the next one a weight of 4, and so on. The global Image-of-Aging score is comprised between −5 and +5 (neutral score at 0); higher scores express more positive views of older people.

The YCVOP, developed for this study, was inspired from the *Children’s Attitudes Toward the Elderly* (CATE) scale (Jantz et al., 1976), the *Children’s Views of Aging* (CVOA) scale (Marks et al., 1985), and a cartoon method designed by Caspi and used in a pilot study (Caspi, 1984). CATE, or parts of it, has been repeatedly used in children between 3 and 12 years (Page et al., 1981; Seefeldt, 1984; Zandi et al., 1990; Middlecamp and Gross, 2002; Cummings et al., 2004); CVOA, or parts of it, was shown appropriate in children between 6 and 16 (Newman et al., 1997; Chorn Dunham and Casadonte, 2009; Flamion et al., 2019); and Caspi’s methodology was applied to children between 3 and 6.

The YCVOP evaluates young children’s views of older people through 11 bipolar pairs of adjectives. Eight of these 11 pairs were extracted from the three original tools (CVOA, CATE, and Caspi’s): *Sad–Happy*, *Slow–Fast*, *Dirty–Clean*, *Sick–Healthy*, *Dull–Exciting*, *Hated–Loved*, *Mean–Kind*, and *Weak–Strong*. Two of these pairs (*Hated–Loved, Mean–Kind*) were considered adequate to probe the warmth aspects, because they replicate terms classified under the warmth aspect of the Stereotype Content Model (Cuddy et al., 2008), and two pairs (*Slow–Fast*, *Weak–Strong)*, were designed to probe physical competence in children’s views (refer to Harter, 1982). Four pairs (*Sad–Happy*, *Dull–Exciting, Dirty–Clean*, *Sick–Healthy*) probe other typical age-related stereotypes in a strongly dichotomized way. Finally, to assess competence in a broader way, we decided to add three pairs of adjectives (*Dumb–Smart, Dependent–Independent, Passive–Active*), inspired from the description of competence in the Stereotype Content Model (Cuddy et al., 2008) and in recent studies in children (Balázs, 2013; Vauclair et al., 2018). The positive and negative ends of each pair were alternated from one pair to the next, respecting the original arrangement in CATE and CVOA. However, the original Likert-type methodology used in CATE and CVOA was transformed into a 25-cm visual analog scale (VAS) approach. We asked each child to express her/his feelings toward old people in general using these 11 bipolar pairs of adjectives.

**FIGURE 1:**
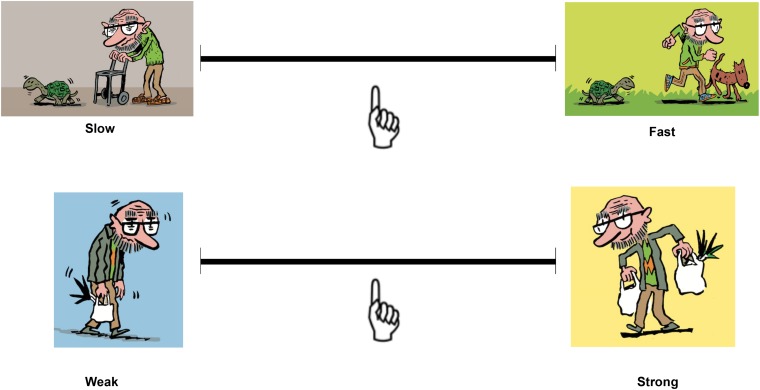
Two examples of the Young Children’s Views of Older People (YCVOP) scale. The child has to slide a handle and point the finger at a position that best describes her/his view of older people on the line between two opposed adjectives, each illustrated by a cartoon.

To make the concepts covered by the adjectives accessible to the kids, a Belgian cartoonist, Philippe Sadzot, illustrated each pair of adjectives by means of small cartoons representing, for instance, strength, kindness, and independence. This method was inspired by Caspi’s work (Caspi, 1984). In the YCVOP, color drawings representing the same old man with different expressions were printed on 10.4 by 14.0 cm (4.1 × 5.6 in) plastic cards and each of them was placed on Velcro^TM^ tape to the extremities of a horizontal plastic line representing the VAS, along which the child had to move a finger-shaped handle/slider (Figure 1). This system is inspired by medical scales used to assess pain (emotional and personal experience) in young children, even as young as 3 years (Cohen et al., 2008). The tool can be handled and manipulated as the child wishes. Before taking the YCVOP, each child was requested to pass a training test using completely different adjective pairs. All children passed the test, confirming they understood the mechanism of the VAS and its possible variations (selection of the extremities, the middle, or a more nuanced choice).

In the current study, the YCVOP was administered to each child in a standardized way. For each bipolar adjective pair, the experimenter asked: “*According to you, an old person is rather (first adjective of the bipolar pair), (second adjective), or somewhere in between?*,” and then let the child move the slider along the VAS. We ran pilot exercises in the same age group, which showed that, for two pairs only (*Dependent–Independent* and *Passive–Active*), additional explanation was needed to ensure fill understanding of the concepts. For these two pairs, we provided the child with the following standard oral explanations: “*The old person must be helped or can do everything on her/his own*” (*Dependent–Independent*), and “*The old person usually does nothing or usually does a lot of things*” (*Passive–Active*). The final position of the slider generated a score (value present at the back of the VAS and not seen by the child) for each pair of adjectives ranging from −50 (extremely negative) to +50 (extremely positive). We calculated a mean global score, also comprised between −50 and +50 (neutral score at 0), for each child (Cronbach’s α = 0.70). Higher scores on the YCVOP indicate more positive views of older people.

#### Parents’ Views of Older People

We assessed the views of each parent on older people using, in sequence, the same open-ended Image-of-Aging question as for their child, and the validated French version (Boudjemadi and Gana, 2009) of the *Fraboni Scale of Ageism-Revisited* (FSA-R). We asked the same external evaluators used for the children’s words to assess the parents’ words. However, we told them to rate the words as coming from adults instead of preschoolers. We reasoned that this distinction would be important since the emotional meaning of words varies with age (Bahn et al., 2017). In our study, following these instructions, the ratings for the same word given by a child or by a parent did not necessarily match (for examples, see Table 3). The inter-rater reliability of external evaluators for the parents’ responses was 0.98. A global Image-of-Aging score was allocated to each parent through the same procedure used for their child.

We selected the FSA-R as the second instrument measuring ageist stereotypes in adults. The FSA-R is a revised version of the original FSA (Rupp et al., 2005), which was shown effective for this purpose in large samples of adults, from 18 to 98 years (Bodner and Lazar, 2008; Bodner et al., 2012). The FSA-R evaluates the level of ageism through 14 statements the subject must evaluate on a Likert-type scale ranging from 1 (strongly disagree) to 5 (strongly agree). Examples of these items are: “*Many old people are stingy and hoard their money and possessions*,” “*I sometimes avoid eye contact with old people when I see them*,” or “*I personally would not want to spend much time with an old person.*” The sum of the statements yields a global score (α = 0.78 in the current study) comprised between 14 and 70 (mid-score, 42). Contrary to the other assessments, for the FSA-R, higher scores reflect higher levels of ageism.

### Data Analyses

We performed all statistical analyses with the 23.0 version of *“Statistical Package for the Social Sciences”* (SPSS; IBM Corp.). Results of continuous variables are provided below as means (*M*) ± standard deviation (*SD*). We analyzed results normality with the Shapiro–Wilk test. Normality was not approved for the children’s Image-of-Aging (*p* < 0.05) and YCVOP results (*p* < 0.01), nor for the parents’ FSA-R results (*p* < 0.01). Therefore, we used non-parametric tests (Spearman’s *r*_*s*_ together with *p* values) to assess correlations between scores on the different scales, and between views of older people within child–parent dyads. The effect of gender on children’s views was measured with Mann–Whitney *U* tests. Power analysis performed with G^∗^Power 3.1 (Faul et al., 2009) indicated a sample size of 84 subjects to reach a power of 0.80 at an *α* < 0.05, with a medium Cohen’s effect size (0.30). The sample size was eventually larger than required (126 instead of 84) because of convenience sampling in the different schools and classes contacted at the same time, and also to account for an approximately 25% expected missing data in the youngest children’s responses.

In the Image-of-Aging questionnaire, 35 children out of 94 who provided at least one word spontaneously mentioned their grandparents or the relations they had with their grandparents. We compared the subsequent YCVOP score of these children with the score of those who did not mention grandparents. The comparison was performed in an exploratory manner using Mann–Whitney *U* tests.

## Results

### Sample Characteristics

The sample was composed of 126 child–parent dyads (Table 1). Children participants (*M* = 4.6 years; *SD* = 1.0) were 46.8% girls and 53.2% boys. The parent sample was composed of 82.5% mothers and 17.5% fathers, with a broad age distribution between 22 and 62 years (*M* = 36.2; *SD* = 5.8). Eighty-nine percent were in a relationship or married and three-quarters had at least undergraduate education. All children and parents were of Caucasian origin.

**TABLE 1:** Participants’ characteristics and views of older people.

Participants characteristics	Children (*n* = 126)		Parents (*n* = 126)	
	*N (%)*	*Mean (SD)*	*N (%)*	*Mean (SD)*
**Age** (years)		4.6 (1.0)		36.2 (5.8)
**Gender**				
Female	59 (46.8)		104 (82.5)	
Male	67 (53.2)		22 (17.5)	
**Marital status**				
Single			4 (3.2)	
In a relationship/married			112 (88.9)	
Separated/divorced			9 (7.1)	
Unknown			1 (0.8)	
**Higher level of education**				
Middle school			14 (11.1)	
High school			17 (13.5)	
Bachelor			52 (41.3)	
Postgraduate			36 (28.6)	
Ph.D.			7 (5.6)	

**Views of older people**		***Mean (SD)***		***Mean (SD)***

Image-of-Aging score (on *n* = 94 children)		0.4 (1.9)		0.6 (1.8)
YCVOP global score		18.8 (19.4)		
FSA-R global score				27.2 (5.6)

*Image-of-Aging is assessed on a scale of −5 (most negative) to +5 (most positive) with a neutral score of 0; YCVOP, Young Children’s Views of Older People, on a scale of −50 (negative opinion) to +50 (positive opinion) with a neutral score of 0; and FSA-R, Fraboni Scale of Ageism-Revisited, on a scale of 14 to 70 representing increasing ageism, with a mid-score of 42.*

### Children’s Views of Older People

The Image-of-Aging and YCVOP scores (Table 2) were not correlated (*r*_*s*_ = 0.15, *p* = 0.14), suggesting these scales measure different attributes. The effect of gender on children’s views in both scales was not significant (Image-of-Aging: *z* = −1.5, *p* = 0.14; YCVOP: *z* = −0.13, *p* = 0.90).

**TABLE 2:** Correlations between the different views of older people in children and one of their parents.

Views of older people	1	2	3	4
1. Children Image-of-Aging				
2. Children YCVOP	0.154			
3. Parents Image-of-Aging	0.008	−0.113		
4. Parents FSA-R	0.014	0.057	−0.213*	

YCVOP, Young Children’s Views of Older People; FSA-R, Fraboni Scale of Ageism-Revisited. The children–parents correlations were analyzed using Spearman’s r_s_ coefficients (given in the table) in 94 dyads (Children’s Image-of-Aging score) or 126 dyads (YCVOP). Statistical significance is indicated by: *p < 0.05.

#### Image-of-Aging Measure

**FIGURE 2:**
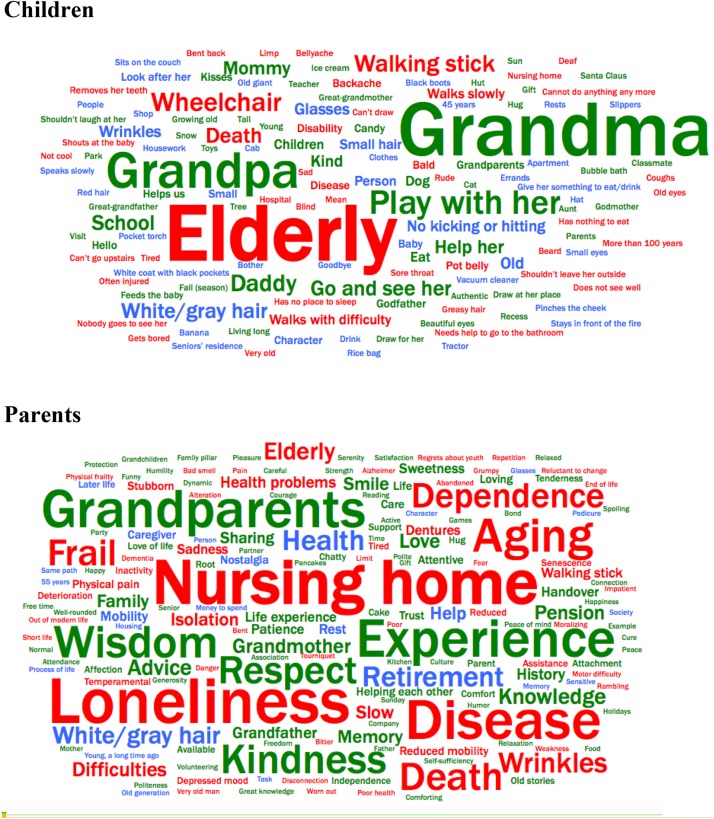
Clouds of words produced by children and parents at the Image-of-Aging question, asking them to cite the first 5 words that came to their mind when thinking of an old person. Clouds were generated by translated versions of the French words cited. The color of each word represents its valence; Red denotes negative words (–5 to –1), green denotes positive words (+1 to +5), and blue denotes neutral words (–0.99 to +0.99). The size of each word is correlated with its frequency (i.e., the more often a word was cited, the larger its size). For instance, in the children’s cloud, *Elderly* is the most frequently cited word (9.4% of all citations) and was given a valence of –2.2 (on a scale of –5 to +5); *Grandma* (9.1%), a valence of +2.9; and *Grandpa* (6%), a valence of +2.5. In the children’s cloud, regardless of the gender stated by the child, the gender was made uniform (to feminine) when the cloud was created, for better visual consistency.

Three-quarters of children (94 out of 126; *M* = 4.7 years; *SD* = 1.0) gave at least one word to describe old people. The 32 children who did not provide any words represent around one third of the 3-year-olds (6 out of 19), one third of the 4-year-olds (13 out of 37), one quarter of the 5-year-olds (12 out of 46), and only one 6-year-old child (out of 24). The mean value of the global Image-of-Aging score was neutral, with a location at 54% of a scale where the most favorable views of older people are at 100%. A total of 135 different words used to describe old people were recorded (Figure 2 and Table 3). Thirty-eight% of these words were evaluated as positive by the external judges, 32% as negative, and 30% as neutral. When the frequency of appearance of the words was incorporated, the percent of positive words was higher: Out of 308 citations, children gave positive words in 47% of cases (44% for words cited in first position), negative words in 32% of cases (38% for words cited in first position), and neutral words in 21% of cases (18% for words cited in first position). This dispersion of positive, negative, and neutral words also emerged from the qualitative analysis of all citations: The most frequently cited word, *elderly* (9.4% of all citations), was judged negative (−2.2) by the external evaluators, but the next three words (*grandma*, 9.1%; *grandpa*, 5.8%; *play with her/him*, 3.6%) were judged positive. The figure of grandparents was prominent: it represented 16% of all citations, and 35 children (37%; mean age, 4.9 years) either cited their grandparents (*grandma* in 12 cases, *grandpa* in 1 case; both in 22 cases) or clearly referred to them during the Image-of-Aging measure. Children who spontaneously evoked their grandparents during the Image-of-Aging had a much higher global Image-of-Aging score than those who did not evoke their grandparents (*M* = 23.7 ± 2.5 *[SEM]* vs. *M* = 13.4 ± 2.8, respectively; *z* = −2.3, *p* < 0.05). These two groups of children did not differ with regard to their gender or age.

**TABLE 3:** The 20 words most often cited by children and parents to describe older people (in descending order) with their occurrence and their assessment scores provided by external evaluators.

Children’s words and their occurrence (%)		Assessment (−5 to +5)	Parents’ words and their occurrence (%)		Assessment (−5 to +5)
Elderly	9.4	–2.2	Loneliness	4.6	–3.1
Grandma	9.1	2.9	Nursing home	4.4	–2.8
Grandpa	5.8	2.5	Disease	3.9	–3.3
Play with her	3.6	3.3	Experience	3.7	4.4
Wheelchair	2.6	–2.2	Grandparents	3.7	3.1
Walking stick	2.3	–1.9	Aging	3.2	–1.1
Daddy	2.3	1.2	Wisdom	3.1	4.3
Go and see her	1.9	3.7	Kindness	2.8	4.4
White/gray hair	1.9	0.1	Death	2.6	–4.3
School	1.9	1.8	Respect	2.6	4.4
Death	1.9	–4.3	Frail	2.4	–1.9
Help her	1.6	1.2	Dependence	2.3	–3.2
Mommy	1.6	3.0	Health	1.8	0.8
Old	1.3	0.3	Retirement	1.6	0.8
Kind	1.3	4.6	Wrinkles	1.6	–1.5
Glasses	1.3	0.0	White/gray hair	1.5	–0.5
Wrinkles	1.3	–0.7	Elderly	1.5	–2.1
Dog	1	1.4	Knowledge	1.3	3.5
Walks with difficulty	1	–2.5	Advice	1.3	4.1
Children	1	1.2	Love	1.1	4.4

*Occurrence of each word is the percentage of all citations giving this word. Assessment was given on a scale from −5 (most negative) to +5 (most positive) with 0 as neutral.*

Overall, words expressing a warm side (e.g., *kind, loved, kisses, hug*) coexisted with those highlighting physical decline (e.g., *walking difficulty, backache, tired, disease).* At an individual level, ambivalent responses, mixing positive and negative words (i.e., words with an evaluated score of >1 or <−1), were noted in one-third of children.

#### YCVOP Measure

On this scale, the distribution of results within the minimal (−48.4) and maximal (+ 50.0) values given was broad. One fifth of the children (25 out of 126) give dichotomous values only, firmly selecting one adjective vs. the opposite adjective in each pair. That outcome was more often observed in 3- and 4-year-olds (in 26% and 30% of these children, respectively) than in 5- and 6-year-olds (in 15% and 8% of these children, respectively). The YCVOP mean value (*M* = 18.8) was on the positive side, located at 68.8% on a scale where 100% represents the most favorable views. Children who spontaneously evoked their grandparents during the Image-of-Aging had much higher YCVOP scores than those who did not evoke their grandparents (*M* = 1.54 ± 0.23 [*SEM*] vs. −0.27 ± 0.23, respectively; *z* = −4.7; *p* < 0.001).

**FIGURE 3:**
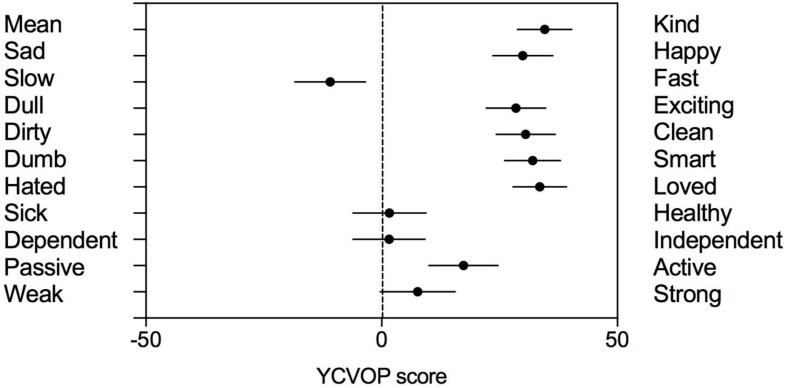
Mean scores of each item of Young Children’s Views of Older People (YCVOP) (⋅) with their 95% confidence intervals (—). The bipolar pairs are presented below in the order they were administered. When the 95% confidence interval does not overlap 0, the result for this adjective pair is significantly (*p* < 0.05) different from 0. Each bipolar pair of adjectives (given in French in the original version) attributed to older people by children was illustrated by a dedicated cartoon (for examples, see Figure 1).

Results of an item by item analysis of the YCVOP (Figure 3) showed some nuances. First, there were significant correlations between the results on the pairs that were grouped under evaluation of warmth (*r*_*s*_ = 0.33, *p* < 0.001) and physical competence (*r*_*s*_ = 0.26, *p* < 0.05). On the other hand, there was no correlation between the three pairs grouped under broader competence, indicating they measure different concepts. Second, 8 out of 11 items resulted in choices that were significantly different from zero: The two pairs assessing warmth traits (*Mean–Kind*, *Hated–Loved*) as well as the *Sad–Happy, Dull–Exciting*, and *Dirty–Clean* pairs, which were all judged highly favorably by the children, with a 78–85% mean weight on the more positive adjective of the pair. With regard to the assessment of abilities and competence, *Dumb–Smart* and *Passive–Active* were judged positively (82% and 67%, respectively), while the *Dependent–Independent* choice was not significantly different from neutral. As for the two pairs probing physical ability, one, *Weak–Strong*, was judged neutral, and the other, *Slow–Fast*, was weighted at 79% toward *Slow.* The *Sick–Healthy* pair was judged in a neutral way. Similar results were observed across the age span of the sample, except for *Slow–Fast*: That was the only choice for which there was a significant correlation with the children’s age (*r*_*s*_ = −0.28, *p* < 0.01), so that older children leaned more toward the *Slow* extremity of the pair. Finally, in accordance with the Image-of-Aging results, ambivalent responses at the individual level were also detected on the YCVOP in a large majority of children: 123 out of 126 respondents evaluated at least one pair of adjectives on the negative side, while assessing many pairs on the positive side. Overall, the Image-of-Aging and YCVOP results together bring out a low-competence, high-warmth stereotype of older people. The low competence arm is mostly represented by low physical capacity and a neutral judgment on the *Dependent–Independent* pair.

### Parents’ Views of Older People

Results on the Image-of-Aging question and the FSA-R scale (Table 2) were significantly correlated (*p* < 0.05) with a Spearman’s coefficient of −0.21, suggesting some similarities between the attributes of the two scales.

#### Image-of-Aging Measure

The mean score of the parents was neutral, located at 56% of the scale. For the 94 parents of the dyads in which children had an Image-of-Aging score, the mean parents’ score was 55%. This value is almost identical to their children’s result (54%). All parents gave at least one word and the majority of them (118 out of 126) gave five words. A total of 181 different words (Figure 2 and Table 3) were recorded on Image-of-Aging. A comparison of the two clouds in Figure 2 shows more agreement amongst parents’ words than amongst children’s words. The most frequent words given by parents differed from those of children (Table 3) but they also comprised both positive (*experience*, 3.7%; *grandparents*, 3.7%; *wisdom*, 3.1%) and negative (*loneliness*, 4.6%; *nursing home*, 4.4%; *disease*, 3.9%) items. Based on all the cited words (614 citations), parents gave positive words in 47% of cases and negative words in 42%. The percent of positive words mirrors the children’s result (47%) but the percent of negative words is slightly higher than the children’s result (32%). The parents’ spontaneous words for old people expressed both a warm side (*kindness, love, smile, sweetness*) and physical decline (*disease, frail, health problems, physical pain, reduced mobility*). The parents’ words of old people also translated increased wisdom (*experience, wisdom, respect, knowledge, advice*) besides sentimental isolation (*loneliness, isolation, sadness*). These wisdom and isolation concepts were not found in the children’s responses. Another difference between children’s and parents’ responses was the prominence of grandparents in the cited words: It was less marked in the parents’ responses (6%) than in the children’s responses (16%). At the individual level, 72% of parents gave both positive and negative words to describe older people.

#### FSA-R Measure

The parents’ mean score was located at 24.1% of the scale where the highest level of ageism is 100%. This result translates a globally low level of ageism.

### Child–Parent Dyads

No significant correlation was found (Table 2) between the children’s views of older people (*n* = 94 for Image-of-Aging and 126 for YCVOP) and the views of their selected parent (Image-of-Aging and FSA-R).

## Discussion

### Elaboration and Value of the YCVOP

In the current study we have developed a tool, the YCVOP, to collect the views of older people in a very young (3- to 6-year-old) preschool population with limited linguistic abilities. This tool allows the assessment of age-related stereotypes in preschoolers. The YCVOP is a semantic differential scale based on pairs of opposed adjectives that incorporates elements of existing scales into a handy, cartoon-illustrated, VAS tool. Its administration was easy and speedy (maximum 20 min), and all children enjoyed its playful, funny character. Some children asked if they could take it again because ‘*it was fun.*’ No child stated that they did not understand the requests or the adjectives and concepts illustrated by the cartoons. The YCVOP internal consistency in the current study was 0.70. Its results were not correlated with those of the Image-of-Aging question (providing five spontaneous words to describe an old person), suggesting these two measures test different aspects of children’s views of older people: stereotypical adjectives for YCVOP, and more personal and emotional associations for Image-of-Aging. One important asset of the YCVOP revealed by the current study is that its completion rate was 100%, whereas the Image-of-Aging was much more challenging and even proved impossible (no word offered) for about one quarter of the children (one third in 3- and 4-year-olds). In addition, the visual aspect of the YCVOP should allow easy reproduction across language barriers.

The YCVOP uses a VAS method, contrary to all semantic differential scales used in previous studies in young children for similar purposes, including the well-known CATE, which were based on Likert-type scales. The VAS approach, combined with its cartoons, offers improved sensitivity (Sinkin-Feldman et al., 1997), better accuracy, and, based on our own pilot tests, excellent comprehension by very young children. Although 25 out of 126 children gave dichotomous responses (pushing the slide onto one extremity of the VAS) on all 11 items of the YCVOP, 13 of these children gave nuanced responses (located between the extremities) on two training exercises, supporting a proper use of the VAS. Dichotomous responses during the actual test may rather reflect the difficulty for very young children (in particular 3- and 4-year-olds) to nuance their view of a person, in this case older people, with regard to two opposed adjectives. Asking the children to verbalize their responses, in addition to sliding the finger-shaped handle, would have been interesting and could be added as a sensitivity tool in future applications of the YCVOP.

The YCVOP has some limitations. For instance, the color pictures used to illustrate each pair of adjectives represent the same old man with different expressions. Because children tend to show less age bias against men than against women (Isaacs and Bearison, 1986), quantitative results of the study along the YCVOP may have been somewhat underestimated. If needed, in future studies, pictures of an old woman could also be used. Moreover, the meanings of the drawings and the associated adjectives may not have been exactly superimposed in the child’s mind. This point was not formally tested in the current study but could be evaluated in future experiments. For example, children may be asked to pick one of several color pictures to fit a particular adjective, or, vice-versa, guess the adjective fitting a color picture. A faultless superimposition between pictures and adjectives would allow subsequent use of pictures only. Finally, the drawings themselves can be thought of as stereotypical and may accentuate old age-related stereotypes (Montepare and Zebrowitz, 2002). However, the point is, the drawings represent a nuanced choice the child has to make between two extremes: What is judged is not the child’s agreement with the stereotype embodied by the drawing but his relative preference toward one or the other side of the bipolar pair of adjectives.

### Children’s Views of Older People

On both the YCVOP and the Image-of-Aging measures, 3- to 6-year-old children’s views of older people were, on average, neutral to positive. This observation may seem in conflict with research showing clear signs of negative views of older people in preschoolers (Kocarnik and Ponzetti, 1986; Seefeldt, 1987) and ageist thoughts toward older people in 3-year-old children (Kwong See and Nicoladis, 2010). Yet, it is in line with recent studies performed in older children, e.g., in a large (*n* = 1151) group of 7- to 16-year-old youngsters in Belgium (Flamion et al., 2019), and in 8- to 12-year-old children in the United States (Robinson et al., 2015). There may be several explanations to this apparent discrepancy.

First, many children in our sample seemed to refer to positive views of their grandparents when they were asked to think of, and give words for, “an old person” (in general). In Image-of-Aging, more than one-third of the respondents cited either *grandma* or *grandpa*, or expressly mentioned their grandparents in other spontaneous statements. The children’s strong preference for the adjectives *loved* and *kind* in the YCVOP differential scales, and for words such as *play with her/him, go and see her/him, draw at her/his place*, or *picks me up at school* in their Image-of-Aging responses, may thus be linked to mental representations of their grandparents. This is not surprising as the most familiar older people in children’s lives are their grandparents (Newman et al., 1997). Strikingly, children who evoked their grandparents in Image-of-Aging gave much more favorable evaluations of older persons on YCVOP than those who did not evoke grandparents. The order in which the tests were performed in our study (Image-of-Aging first) may thus have primed some children to think about their own grandparents, rather than about older adults in general, and may have decreased the amount of negative age bias revealed by YCVOP, administered second. Strong tempering effects of warm relations with grandparents on age-related stereotypes from early childhood throughout adolescence have been shown in previous studies with older children (Lichtenstein et al., 2005; Flamion et al., 2019).

Second, measures that collect children’s views of older people in an explicit way, such as the YCVOP, may induce apparently more favorable views than those elicited by implicit measures, at least in school-age children (Babcock et al., 2016; Mendonça et al., 2018). This difference may also apply to preschoolers, although not demonstrated so far. Contrary to Kwong See and Nicoladis (2010) experiment in 3-year-olds, our study only partially relied on more spontaneous expressions of views of older people (Image-of-Aging remains an explicit measure because children are explicitly asked to describe “an old person”). Our study, like other studies using explicit measures, may have somewhat overshadowed negative views of older people.

### Ambivalent Views of Older People

Children gave a lot of positive terms describing older people’s warmth in Image-of-Aging, and several semantic differential pairs of YCVOP yielded highly positive results, including the two pairs assessing warmth. Nevertheless, some negative aspects emerged. In particular, preschoolers in our study identified the inevitable decline in physical capacities inherent to older age. For instance, in Image-of-Aging, children spontaneously cited terms like *wheelchair*, *walking stick*, *walks with difficulty, disability, cannot do anything any more* or *does not see well*. Similarly, in YCVOP, they clearly judged older persons *slow* rather than *fast*, and midway between *healthy* and *sick*, and between *strong* and *weak*. This contrasts with the highly favorable evaluations of most other adjective pairs. A similar, globally positive perception of older adults accompanied by lower scores on either the *weak–strong* (Caspi, 1984) or the *slow–fast* (Newman et al., 1997) attributes was observed long ago in 3- to 6- and 8- to 11-year-old children, respectively. In the current study, the competence of older people to be *independent* was also judged doubtful.

The pattern emerging from all these views of older people is thus a form of ambivalence: Warmth on the one hand associated with physical decline and sizeable dependence on the other hand. Other features of “broader competence,” i.e., smartness and level of activity, were judged positively. That ambivalence (warmth vs. physical decline and dependence) mimics the typical “doddering but dear” phenotype (Cuddy and Fiske, 2002), which is frequent in young adults’ views of older people. Older people are most often described and categorized by younger adults as less competent (less responsible, less independent, and less ambitious) but warmer (more friendly) than middle-aged or young adults. These views may lead to a distinct type of paternalistic prejudice against older people: pity and sympathy. That phenotype is a reflection of the Stereotype Content Model (Fiske et al., 2002), which suggests that most stereotypes contain both negative and positive beliefs along multiple dimensions. The Stereotype Content Model developed by Fiske et al. (2002) assumes that perceivers’ stereotypes, emotional prejudice, and behavioral reactions toward members of specific groups are driven by a combination of competence (“*How well can this person enact those intentions?*”) and warmth (“*How friendly and trustworthy are this person’s intentions?*”). Parents in the current study indeed offered a high-warmth profile for older people, although, on the competence side, they focused on low physical capacity and dependence (like their child) rather than on a broad range of low competence.

In summary, the ambivalent picture of older persons that emerges from preschoolers’ responses is similar to their parent’s picture and evokes a low-competence, high-warmth stereotype, even though low competence is mostly described as low physical capacity and some dependence.

### Child–Parent Dyads

Our study also revealed that preschool children’s views of older people were not related to the views of their selected parent, despite a similar mean global score on Image-of-Aging in children and parents. This seems to contrast with Degner and Dalege (2013)’s conclusion that child–parent intergroup attitudes are related throughout childhood and adolescence. However, we note that all the studies integrated in Degner and Dalege’s meta-analysis were based on ethnicity, religion, and body weight intergroup attitudes, not on age-related views. Furthermore, the authors suggested that the weakest child–parent correspondence found in early ages is partly due to the divergent methods used to evaluate intergroup attitudes between parents and younger children. A similar criticism could apply to our study since only one of the two measures, the Image-of-Aging, was identical between the child and parent evaluations. However, the responses on that instrument showed a clear difference between children and parents: The preschoolers provided more personal and emotional associations (which was confirmed by a lack of correlation between the results on their Image-of-Aging and YCVOP), while the parents tended to adopt global stereotypes (confirmed by the correlation between the results on their Image-of-Aging and FSA-R, and by a higher internal agreement within the parents’ words than within the children’s words on Image-of-Aging).

Our study was limited to a single parent’s interview, generally the mother, as expected based on availability. Although this gender selection may limit generalizability, we would like to point out that any similarity in views within a child–parent dyad was expected to be higher with the mother, because, even today, most families follow traditional settings in which mothers tend to spend more time with their children than fathers. A study found slightly higher mother–child than father–child similarity in attitudes toward ethnic minorities (Jaspers et al., 2008). Degner and Dalege (2013), in their large meta-analysis, found that parent gender had no significant moderation effect on child–parent correlations regarding the expression of intergroup attitudes. All in all, we can conclude that the absence of mother–child correlation in views on older people is relevant for parent–child attitudes. This observation may reveal a true individualism of the young (<7 year-old) child’s perception of older people. This is in line with the recent observations of Lineweaver et al. (2017). These authors found that children’s ageist stereotypes were related to those of their parents from 9 years on, and that the older the child, the stronger these child–parent common stereotypes (Lineweaver et al., 2017). Their study found no child–parent correlation in 6- to 7-year-olds. In very young children, parental influence on age-related stereotypes may be intermingled with, and overcome by, other factors such as media and peer group influence, or regular contacts with grandparents (Flamion et al., 2019).

### Implications for Intergenerational Programs

Several school curricula, recognizing the importance of acting early against age-related negative stereotypes, have started to incorporate intergenerational programs designed to improve children’s views and attitudes toward old people, with some success (Christian et al., 2014; Park, 2015). The results of our study could inspire such programs in preschool children. These programs could focus, for instance, on the main negative stereotype identified in this age group in the current study, i.e., that older people lack physical capacity and become slower and more dependent. Real examples of, and contact with, healthy-aging, physically active older adults could change the low competence profile attributed by these children to older people in general. Gualano et al. (2018), in a recent review of intergenerational programs, observed that co-location of pre-schools and nursery homes in the same building brought some logistical advantages when it came to intergenerational contacts; yet, such a setting could accentuate the idea that older people are frail and dependent. The results of our study would also support the involvement of grandparents in intergenerational programs. A good example was provided by Thompson and Weaver (2016) who interviewed 944 students aged 13 to 19 having attended or not a regional intergenerational program when they were in fourth grade. That program intensified their contacts with older adults, in many cases one of their grandparents. This experience had an obvious favorable impact on the students’ views of older people 5 to 9 years later.

### Limitations

All children and parents in this study were Caucasian Whites, which limits generalizability to other ethnic contexts. Data on the amount and quality of intergroup contact between preschoolers and old adults, including their own grandparents, were not collected, which means this factor could not be analyzed as a potential modulator of children’s views.

## Conclusion

Using explicit measures partly developed for this study, a globally favorable view of older people was found in a sample of 126 three- to six-year-old preschool children from urban and rural regions of Belgium. The warmth and smartness traits of old people came out clearly. However, some of the children’s views were distinctly stereotypical and negative, especially about older people’s physical abilities and health. Overall, the preschoolers’ responses, like their parents’ responses, were in line with a low-competence, high-warmth stereotype that evokes the Stereotype Content Model highlighted in previous studies with young adults and 6- to 15-year-old children (Fiske et al., 2002; North and Fiske, 2012; Vauclair et al., 2018). In our study, children’s views of older people did not correlate with their parent’s views but were significantly more positive in children who spontaneously evoked their grandparents when asked to think of an old person. Insisting on older people’s competence and integrating grandparents in future intergenerational programs designed for preschool children appear valuable targets.

## Data Availability Statement

The datasets generated for this study are available on request to the corresponding author.

## Ethics Statement

The studies involving human participants were reviewed and approved by the Ethics Committee of the Faculty of Psychology at the University of Liège reviewed and approved the research procedures with human subjects. Written informed consent to participate in this study was provided by the participants’ legal guardian/next of kin.

## Author Contributions

AF designed the study, supervised the data acquisition, performed the statistical analyses, and wrote the manuscript. PM revised the article. LJ and NH acquired the data. SA supervised the study and provided advice at all steps.

## Conflict of Interest

The authors declare that the research was conducted in the absence of any commercial or financial relationships that could be construed as a potential conflict of interest.
